# How do extrinsic cues influence consumers’ online hotel booking decisions? An event-related potential experiment

**DOI:** 10.3389/fpsyg.2022.990640

**Published:** 2022-11-29

**Authors:** Ping Feng, Jingqiang Wang, Xin Ding, Cong Li, Fumei Guo, Xinyi Ding

**Affiliations:** ^1^College of Tourism, Huaqiao University, Quanzhou, China; ^2^School of Hotel and Tourism Management, Hong Kong Polytechnic University, Kowloon, Hong Kong SAR, China

**Keywords:** online review, brand familiarity, booking decisions, P200, LPP, N400

## Abstract

Booking decision is a typical decision-making behavior in hospitality, while the neural processing of it is still unclear. To address this issue, with the help of event-related potential (ERP), this work uncovered the neural mechanism of the influence of two extrinsic cues, namely, brand familiarity (familiar vs. unfamiliar) and online reviews (positive vs. negative) on online hotel booking decisions. Behavioral results indicated that the booking rate under the condition of positive reviews was higher than that of negative reviews. In addition, the response time in the case of familiar brands was longer than that of unfamiliar brands. ERP results showed that the P200 amplitude of familiar brands was smaller than that of unfamiliar brands, while for the late positive potential amplitude, the opposite was the case. It is suggested that in the early stage of cognitive processing, unfamiliar brands evoke more automatic and unconscious attention while in the later stage, familiar brands attract more conscious attention. This study also found that the N400 amplitude of negative online reviews was larger than that of positive online reviews, indicating that negative stimuli can result in a larger emotional conflicts than that of positive stimuli. This study provides new insights into the neural mechanism of online booking decisions in the hospitality.

## Introduction

Event-related potential (ERP) is the electrophysiological brain signals associated with cognitive and emotional responses to an event (Herring et al., 2011). Its formation mainly depends on the weak potential difference produced by the discharge of brain neurons (Wang et al., 2016). To be specific, the weak electroencephalogram (EEG) signals elicited by numerous stimuli are superimposed to remove white noise, and then a series of relevant ERP components are obtained. With the rapid development in the interdisciplinary fields of cognitive neuroscience and marketing, ERP has been increasingly adopted to investigate the decision-making behaviors of consumers.

However, booking decisions, a typical decision-making behavior in hospitality, has rarely been explored by ERP. The plethora of previous research on booking decisions were adopted the traditional self-report approach, which led to two research gaps. First, the traditional self-report approach did not open the “black box” of consumers. In contrast, ERP can gain better insights into individuals’ cognitive-processing and decision-making at the brain level, thus revealing the neural mechanism underlying booking decisions (Fugate, 2007; Dimoka et al., 2010). In addition, the data obtained with self-report method was often blamed for subjective biases in recent years (Kuan et al., 2014), because consumers might not be aware of their real cognitive and psychological processes, and even suppress their real attitudes, feelings and behaviors at sometimes. By contrast, ERP can track the decision-making process in consumers’ brain in real-time by recording the scalp electrical potentials related to specific events, which is more objective and convincing. Therefore, it is time to adopt ERP to disclose the neural activities of consumers and the formation mechanism of their booking decisions.

Consumers usually make booking decisions based on product-related cues, so as to reduce the uncertainty and risks (Casaló et al., 2015a; Agag and El-Masry, 2016; Shin et al., 2018) caused by the intangible nature of hotels (El-Said, 2020). These cues have been dichotomized into intrinsic and extrinsic cues (Olson and Jacoby, 1972). Intrinsic cues refer to the direct physical attributes of products, such as color and texture (Richardson et al., 1994), whereas extrinsic cues refer to the indirect properties of products, such as brands (i.e., brand familiarity, brand image), online reviews, and enterprise reputation (Leavitt, 1954; Griffith and Gray, 2002). Consumers will tend to utilize intrinsic cues if buying functional products, while they will prefer using extrinsic cues if purchasing hedonic products (Maheswaran and Chaiken, 1991; Miyazaki et al., 2005). Given the hedonic attributes of hotel products and services, consumers will rely more on extrinsic cues.

Online reviews and brand familiarity are major extrinsic cues commonly adopted by consumers (Wen et al., 2021). Online reviews are evaluations posted on diverse online platforms by consumers who have used the products or services. As reported by Tnooz (2014), a global PhoCusWright survey showed that over 80% of consumers would read numerous reviews before their final hotel reservation, and 53% would not book hotels without reviews. Brand familiarity refers to “the number of product-related experiences that have been accumulated by the consumers” (Alba and Hutchinson, 1987, p. 411). The product-related experiences includes direct experience (e.g., advertising exposures and information search) and indirect experience (e.g., interactions with salespersons, choice and decision making, purchasing, and product usage). Brand familiarity is a quality signal of brands that can increase certainty and confidence in consumer decisions (Erdem et al., 2008; Foroudi, 2019). Compared to consumers exposed to unfamiliar brands, those exposed to familiar brands respond more positively (Griffith and Gray, 2002).

Although some studies have already discussed the effect of these two cues on online reservations (Wen et al., 2021), most of them concentrate on booking intentions rather than booking decisions. However, there is a real gap between intentions and actual booking behaviors as there are significant differences in their influencing factors and mechanisms of formation (Wang and Li, 2022). Moreover, most of them center on addressing the “what” questions— “what are the effects of the two cues,” rather than the “how” questions—“how do the two cues impact the online booking.”

To bridge the aforementioned research gaps, with the help of ERP, this work examines the effect of brand familiarity (familiar vs. unfamiliar) and online reviews (positive vs. negative) on consumers’ booking decisions in the brain aspect to uncover their underlying neural mechanism. The findings are expected to deepen the theoretical understanding of online booking decisions.

## Literature review and hypothesis development

### Cue utilization theory

Cue utilization theory points out that products convey a series of cues (Cox, 1967), and consumers can apply these cues to evaluate the quality of products and services before making final purchase decisions (Wang et al., 2016; Wen et al., 2021). In hospitality, previous research consistently suggests that the cues, such as user-generated photos (Filieri et al., 2021), product price (Wen et al., 2021), third-party information (Akdeniz et al., 2013), and review rating (Baek et al., 2012), play important roles in consumers’ booking decisions, especially in the online environment.

According to the cue utilization theory, cues are diagnosticity, which refers to their “accuracy and reliability in differentiating the product from its alternatives” (Wen et al., 2021, p. 2). Cues can be categorized as high-scope and low-scope according to their diagnosticity (Purohit and Srivastava, 2001). High-scope cues are perceived to be more credible as they are stable and not easy to be changed, while low-scope cues are unstable and easy to be changed (Purohit and Srivastava, 2001). Existing research argues that consumers consider a few key product cues including high- and low- scope cues together rather than isolate each of them when they search for products’ information before making final decisions (Shah and Oppenheimer, 2008; Tanford and Kim, 2018).

Brand familiarity was regarded as the high-scope cue as it could bring positive brand associations and attitudes (Dawar and Parker, 1994). However, recent research found that brand familiarity is actually a low-scope cue relative to online reviews (Wen et al., 2021). In the present study, online reviews will be discussed as a high-scope cue, while brand familiarity will be regarded as a low-scope cue.

### Event-related potential method and event-related potential components

Event-related potential is the only brain imaging method that can directly monitor neural activities to reflect neurophysiological changes during event cognition (Wang et al., 2020), and therefore, it can open the “black box” in the brain of consumers. In recent years, the decision behaviors of consumers gained increasing attention from researchers in the interdisciplinary fields of cognitive neuroscience and management. For instance, in a pioneering study, Jing et al. (2019) examined the effect of price and quantity promotion on hedonic purchase behaviors by ERP. Shang et al. (2020) uncovered the neural mechanism underlying purchase intention bias during online shopping festivals. Studies mentioned above indicated that it is feasible and beneficial to apply ERP to the research of consumer decision-making behaviors. However, to our knowledge, this research stream is still exploratory, and studies are rare that examine the neural mechanism underlying the influence of extrinsic cues on consumers’ booking decisions. To fill this gap, we attempt to identify how the two cues, brand familiarity and online reviews, take effect from the cognitive neuroscience perspective by adopting three typical ERP components that have been frequently investigated in previous decision neuroscience research, including P200 closely related to early automatic and conscious attention, late positive potential (LPP) associated with later conscious and emotional assessment, and N400 closely related to cognitive conflicts.

### Event-related potential hypotheses

#### P200

P200, an early positive-going component that peaks at around 200 ms after stimulus onset and principally distributes over the posterior scalp (Olofsson et al., 2008), has been concluded to reflect the early automatic and attention arousal (Carretié et al., 2001; Lijffijt et al., 2009). Accordingly, the P200 amplitude has regarded as a typical indicator of the allocation of attentional resources in the early stage of cognitive processes, with a larger P200 amplitude indicating more early attentional resources (Ma et al., 2018; Alzueta et al., 2019). A wealth of research has verified this finding in various scenarios. For instance, Ma et al. (2018) found that the most-preferred products can elicit a larger P200 amplitude than the least-preferred products because they evoke more early attention. Jing et al. (2019) indicated that deep discounts automatically attracts more attention resources than shallow discounts, and therefore lead to a higher P200 amplitude.

Ozkara and Bagozzi (2021) reported that the mental process of making decisions for unfamiliar brands is automatic and unconscious. That is, unfamiliar brands can attract more automatic attention in the early stage of cognitive processes. Moreover, previous research asserted that negative stimuli could automatically recruit more attention and thus elicit a larger P200 amplitude than positive and neutral stimuli (Carretié et al., 2001; Jin et al., 2018; Wang et al., 2020). Compared with familiar brands, unfamiliar brands are negative stimuli and will arouse consumers’ vigilance and further attract their attention. Therefore, we suppose that:

H1: The P200 amplitude of familiar brands is smaller than that of unfamiliar brands.

#### Late positive potential

The LPP is a positive voltage that belongs to P3 family, and its amplitude peaks at around 300 ms after stimulus onset. The difference between LPP and P300 is that LPP has an extended onset time and a more centrally distribution over time (i.e., onset around 300–700 ms and posterior scalp maximum) (Luck, 2014, p. 107). LPP typically reflects attention allocation and time-specific emotional response to stimuli (Hajcak et al., 2006). For instance, appetitive pictures attracted local attention and aroused more positive emotions, thus evoking a larger LPP amplitude compared to neutral stimuli (Gable and Harmon-Jones, 2010). Ma et al. (2018) argued that more-preferred products elicited a larger LPP amplitude than less-preferred products as they aroused a positive emotion.

Recent research argued that the mental process of making decisions for familiar brands is conscious (Ozkara and Bagozzi, 2021). In other words, familiar brands attract more attention in the late stage of cognitive processes. In addition, the research on brand choice indicated that a familiar brand has a greater chance of being selected by consumers than an unfamiliar brand (Andsager and Drzewiecka, 2002) since familiarity strengthens their attachment (Hammitt et al., 2009). In the current research, compared to unfamiliar brands, familiar hotel brands attract more attention and are more emotionally motivated. Therefore, we speculate:

H2: The LPP amplitude of familiar brands is larger than that of unfamiliar brands.

#### N400

N400 is the best-studied language-related negative-going component, which occurs approximately 300–500 ms after stimuli and is mainly found in the central and frontal area of the scalp (Kutas and Hillyard, 1980). Although the N400 is originally discovered in the study of semantic conflicts and lexical violations (Kutas and Hillyard, 1980), most recent research has demonstrated that it also reflects non-semantic conflicts, such as cognitive and emotional conflict (Mai et al., 2004; Ma et al., 2008; Taake et al., 2009).

In fact, researchers in consumer neuroscience have argued that N400 is sensitive to the violations of our knowledge about the world (Huang et al., 2014). In other words, it reflects the deviation between exposed and acceptable information, with a smaller N400 amplitude representing higher acceptability of exposed information (Zhang et al., 2019a). In the current research, compared to unfamiliarity, familiarity activate consumers’ attachment (Hammitt et al., 2009) and reduce the perceived uncertainty and risk of decision-making (Fuchs and Reichel, 2011). Given that, it can be inferred that familiarity brands are more acceptable than unfamiliar brands. Therefore, we suggest the following hypothesis:

H3: The N400 amplitude of unfamiliar brands is larger than that of familiarity brands.

Herbert et al. (2008) found that the valence of adjectives influences the N400 amplitude, and a more pleasant adjective corresponds to a smaller N400 amplitude. This finding has been supported by subsequent research. For instance, De Pascalis et al. (2009) discussed the impact of emotional processing on the N400 amplitude and reported that negative words elicited a larger N400 wave than positive words. In addition, negative reviews are associated with uncertainty, risk, and even threat, which will increase consumers’ emotional conflict than positive reviews, and thus a larger N400 amplitude. Taken together, we suppose that:

H4: The N400 amplitude of negative online reviews will be larger than that of positive online reviews.

According to previous research, N400 is sensitive to every stimuli that violate expectations. For instance, Rasmussen (2007) argued that unattractive-positive word pairs elicit larger N400 amplitude compared with unattractive-negative word pairs.

Wang et al. (2016) suggested that the N400 amplitude of inconsistent cues was larger than that of consistent cues. Baetens et al. (2011) indicated that an increased N400 amplitude is sensitive to the violation of expectations based on the social impression of an individual. In the current research, familiarity is a signal of product quality, and it can activate the attachment of consumers to brands. In other words, individuals naturally expect familiar brands with positive reviews; if familiar brands receive negative reviews, this violation may result in greater cognitive and emotional conflicts and thus elicit a larger N400 than in the case of familiar brands receiving positive reviews. Similarly, individuals normally associate unfamiliar brands with uncertainty and risk; if unfamiliar brands receive positive reviews, the violation may also result in greater emotional conflicts and thus elicit a larger N400 than under the condition of unfamiliar brands receiving negative reviews. Therefore, the following hypotheses are thus put forth:

H5: There is an interaction effect between the online review and brand familiarity on N400 amplitude.

## Methodology

### Experimental design

With the help of questionnaires and ERP technology, this research conducted a 2 (brand familiarity: familiar vs. unfamiliar) × 2 (online review: positive vs. negative) within-subject design. To be specific, we used questionnaires to manipulate the two levels of these two variables, and the detailed process was introduced in Section 3.3 “Materials.” Then, subjects were invited to participate in the ERP experiment. During the experiment, a brand name and a keyword of online reviews were presented in turn at the center of the computer monitor. Subjects decided whether they would like to book a hotel according to the two specific cues by pressing “F” (to book) or “J” (not to book) on the keyboard.

### Participants

In the current experiment, 24 right-handed healthy undergraduate students (14 females, 10 males; *M*_age_: 21.30 years) with similar demographic backgrounds were recruited. All participants had online booking experience in the past 12 months and signed informed consent forms before taking part in the formal experiment and were given cash as compensation upon experiment completion. Data from one male participant was excluded due to the experimental program breakdown when it was his turn and the data from 23 participants (13 females, 10 males) were retained for analysis. The present study was approved by the Ethics Committee of the Neurotourism Laboratory at Huaqiao University.

### Materials

To determine the standard for high versus low brand familiarity, as well as the standard for positive versus negative online reviews, a couple of questionnaires were conducted. The manipulation of brand familiarity is similar to that by Park and Stoel (2005) and Wen et al. (2021), where stimulation of familiar brands showed with existing familiar brands, and stimulation of unfamiliar brands showed with fictitious brands. The specific steps are as follows. First, 10 existing familiar brands and 10 fictitious brands were constructed. Then, 38 subjects were invited to choose the five most familiar brands (Table 1). The results showed that the five most familiar brands were “Home Inn” (97.37%), “7 Days Inn” (89.47%), “HanTing hotel” (86.84%), “Super 8 hotel” (84.21%), and “JinJiang Inn” (80.74%). Among them, “Jinjiang Inn” has a Chinese name composed of four characters, which is different from the other most familiar brands, whose Chinese names are only composed of two characters. In addition, the Chinese name of the brand ranking sixth (“GreeenTree Hotel”) is also composed of four characters. Therefore, “Hotel Ku 6” (44.74%) took the place of “Jinjiang Inn” and “GreeenTree Hotel” in the formal experiment. The five most unfamiliar brands were “Lansen,” “XiuYa,” “XiangYue,” “YanSha,” and “SaiNa” and their chosen rates were all zero. Consequently these 10 brands were used in the formal experiment.

**TABLE 1:** Familiarity rank of the existing and fictitious brands.

Existing brands	Rate of being chosen	Fictitious brands	Rate of being chosen
Home Inn	97.37%	Yi He	7.89%
7 Days Inn	89.47%	Sunshine	5.26%
HanTing hotel	86.84%	Shi Ya	2.63%
Super 8 hotel	84.21%	Fu Run	2.63%
JinJiang Inn	84.21%	Xin Yue	2.63%
GreenTree hotel	52.63%	Lan Sen	0%
Hotel Ku 6	44.74%	XiuYa	0%
Motel	31.58%	XiangYue	0%
Thank U	21.05%	YanSha	0%
Hilnn	13.16%	SaiNa	0%

For online reviews, a complete sentence is not suitable since the ERP experiment requires the stimuli to be as simple and short as possible. Therefore, according to the six aspects of hotel reviews (i.e., location, service quality, environment, room quality, cost performance, and general feeling) (Wen et al., 2021), we extracted 30 positive keywords and 30 negative keywords from real online reviews on Ctrip (one of the major online booking platforms in China). Then, 61 participants were invited to judge whether these keywords matched the six aspects of hotel reviews and whether they were understandable. Subsequently, only 12 positive and 12 negative keywords were retained, and they are all composed of four characters in Chinese (Table 2). Moreover, 33 subjects were invited to score the valence of these 24 keywords on a scale from 1 to 9 (1 = negative, 5 = neutral, 9 = positive). The results showed that there was a significant difference between the positive and negative keywords (*p* < 0.01), indicating that online reviews were manipulated successfully.

**TABLE 2:** Keywords from real online reviews for the experiment.

Aspects of hotel reviews	Positive keywords	Negative keywords
Location	Convenient location; Close to the subway	Inconvenient location; Far from the subway
Service quality	Good service; Considerate service	Bad service; Slow check-in
Environment	Clean; Quiet	Dirty; Noisy
Room quality	Comfortable bed; Good lighting	Poorly qualified bed; Dark and damp
Cost-performance	Cost-effective; Cheap	Cost-ineffective; Expensive
General feeling	Pleasant; Satisfied	Not recommend; Dissatisfied

### Procedure

**FIGURE 1:**
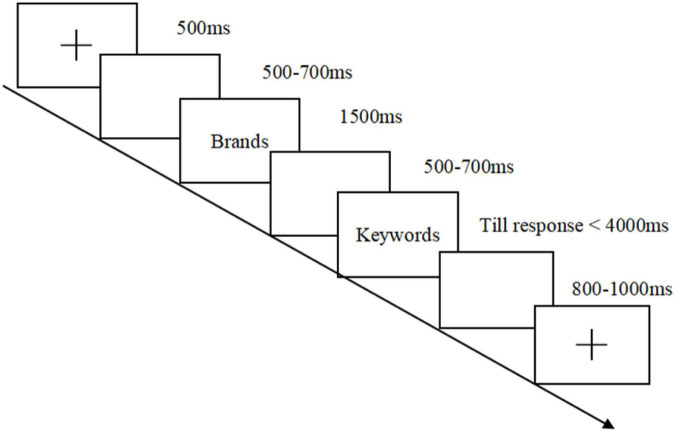
Experimental task: Participants were instructed to make decisions about whether to reserve a hotel according to brand familiarity and online review.

All stimuli were presented and all triggers were recorded via the E-Prime 2.0 software. Each participant was brought to a soundproof neuroscience laboratory and sat in a chair 90 cm away from a 17-inch LCD monitor (pixels: 1280 × 1024; refresh rate: 60 Hz; RGB: 190, 190, 190). At the start of the experiment, the participants were given instructions about the experimental task. On each trial in the formal experiment, a fixation (500 ms), a blank (500–700 ms), a target stimulus of a brand name (1500 ms), a blank (500–700 ms), and a target stimulus of keywords of online review (disappearing either after 4,000 ms or when participants pressed the button) were presented sequentially (see Figure 1). The participants were instructed to make their booking decisions using a keypad (by pressing the button of “F” or “J”) as soon as possible when the keyword was presented. In this experiment, the target stimuli included five familiar and five unfamiliar brands and twelve positive and twelve negative keywords. They were shown in a random order (60 trials under each condition) and constituted 240 trials. To avoid the cognitive fatigue of the participants, the 240 trials were divided into four blocks, and each block was followed by a break. The participants practiced enough before the formal experiment.

### Electroencephalogram recordings and processing

Electroencephalogram (EEG) data were continuously recorded (band pass filter: 0.05–100 Hz; sampling rate: 1000 Hz) with a Neuroscan-64 Synamp2 Amplifier and an electrode cap with 64 Ag/AgCl electrodes mounted according to the extended international 10–20 system. The online reference was on the left and right mastoid, and the forehead location was used as the ground. A vertical electrooculogram (EOG) was recorded from infra-orbital and supra-orbital electrodes placed directly above and below the left eye. A horizontal EOG was recorded from electrodes at the outer canthi of both eyes. Electrode impedances were maintained below 5 kΩ.

Data were transformed using the bilateral mastoid process for reference when processed offline. Data containing artifacts such as blinking, eye movements, and myoelectricity were rejected. After preprocessing the data, ERPs were computed separately for each participant in each experimental condition in 1,000-ms epochs starting 200 ms before stimulus onset (–200 to 0 ms baseline). The rejection criterion was ±100 μV.

Based on the visual observation of grand average waveforms and relevant studies (Wang et al., 2016, 2022; Zhang et al., 2019b), the P200 and LPP were used to analyze the cognitive processing of brand familiarity in the stage of hotel brands, and N400 was used to analyze the cognitive processing of online reviews and booking decisions in the stage of keywords. Regarding P200, the time window of 100–300 ms was chosen to evaluate its mean amplitude to identify neural activities related to brand familiarity. Six electrodes were selected for statistical analysis: F1, FZ, and F2 (frontal area); FC1, FCZ, and FC2 (frontal-central area). In conceiving of LPP, the time window of 300–500 ms was chosen to evaluate its mean amplitude to identify neural activities related to brand familiarity. Six electrodes were selected for statistical analysis: CP3, CPZ, and CP4 (central-parietal area); P3, PZ, and P4 (parietal area). In terms of N400, we referred to the time window of 300–400 ms to assess its mean amplitude to obtain neural activity related to booking decisions. Nine electrodes were included for analysis: F1, FZ, and F2 (frontal area); FC1, FCZ, and FC2 (frontal-central area); and C1, CZ, and C2 (central area). The behavioral data (i.e., the ratio of online booking, and the response time) and accompanying ERP data were examined via SPSS 17.0. Results of repeated measure ANOVAs were adjusted using the correction of Greenhouse and Geisser (1959) to the degrees of freedom. Partial eta-squared values (η^2^) were reported to demonstrate the effect sizes in ANOVA models (Cohen, 1977), and *p*-values were reported for factors with more than two levels.

## Results

### Behavioral results

A 2 (brand familiarity: familiar vs. unfamiliar) × 2 (online review: positive vs. negative) repeated measures ANOVA was performed regarding participants’ response time and booking rate. For response times, a marginally significant main effect of online review [*M*_positive_ = 1078.787 ms, *SE* = 62.632; *M*_negative_ = 999.723 ms, *SE* = 53.379; *F*(1, 22) = 3.948, *p* = 0.060, η^2^ = 0.152] and a significant main effect of brand familiarity were identified [*M*_familiar_ = 1065.109 ms, *SE* = 59.244; *M*_unfamiliar_ = 1013.401 ms, *SE* = 52.142; *F*(1,22) = 5.385, *p* < 0.05, η^2^ = 0.197]. However, we did not find the interaction effect of brand familiarity and online review. For booking rate, a significant main effect of online review was observed [*M*_positive_ = 0.996, *SE* = 0.015; *M*_negative_ = 0.020, *SE* = 0.010; *F*(1,22) = 3165.277, *p* < 0.01, η^2^ = 0.993]. However, neither a significant main effect of brand familiarity nor an interaction effect of brand familiarity and online review were observed (*ps* > 0.05).

### Event-related potential results

#### P200

**FIGURE 2:**
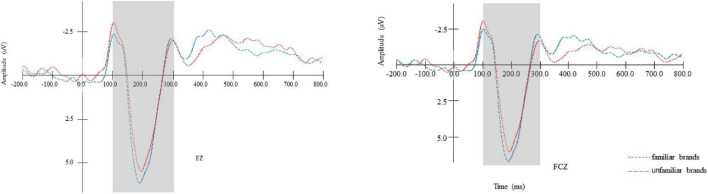
The P200 amplitude comparison of the brand types (familiarity brands vs. unfamiliarity brands) in representative electrodes (FZ and FCZ).

The results of a 2 (brand familiarity: familiar vs. unfamiliar) × 6 (electrodes: F1, FZ, F2, FC1, FCZ, and FC2) repeated measures ANOVA showed a significant main effect of brand familiarity [*F*(1,22) = 6.283, *p* < 0.05, η^2^ = 0.222], indicating that in the early stage of information processing, a larger average P200 amplitude was elicited in the unfamiliar brands condition (*M*_unfamiliar_ = 7.479, *SE* = 0.550) than in the familiar brands condition (*M*_familiar_ = 6.473, *SE* = 0.499), as seen in Figure 2. Therefore, H1 was supported. Meanwhile, we also found a significant main effect of electrodes [*F*(5,18) = 9.281, *p* < 0.05, η2 = 0.279]. To be specific, the P200 amplitude was the largest at FCZ and the smallest at F1. However, the interaction effect between brand familiarity and electrodes was not observed (*p* > 0.05).

#### Late positive potential

**FIGURE 3:**
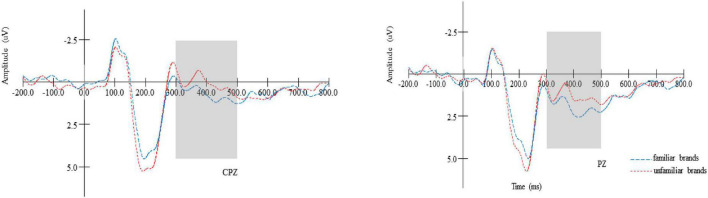
The LPP amplitude comparison of the brand types (familiarity brands vs. unfamiliarity brands) in representative electrodes (FZ and FCZ).

The results of a 2 (brand familiarity: familiar vs. unfamiliar) × 6 (electrodes: CP3, CPZ, CP4, P3, PZ, and P4) repeated measures ANOVA showed a marginally significant main effect of brand familiarity [*F*(1,22) = 4.293, *p* = 0.051, η^2^ = 0.177], suggesting that, in the later stage of information processing, a larger average LPP amplitude was elicited in the familiar brands (*M*_familiar_ = 2.052, *SE* = 0.532) condition than in the unfamiliar brands condition (*M*_unfamiliar_ = 1.271, *SE* = 0.343), as seen in Figure 3. Therefore, H2 was supported. Meanwhile, we also found a significant main effect of electrodes [*F*(5,18) = 11.128, *p* < 0.01, η^2^ = 0.777]. The LPP amplitude was the largest at P4 and the smallest at CPZ. However, an insignificant interaction effect between brand familiarity and electrodes was observed (*p* < 0.05).

#### N400

**FIGURE 4:**
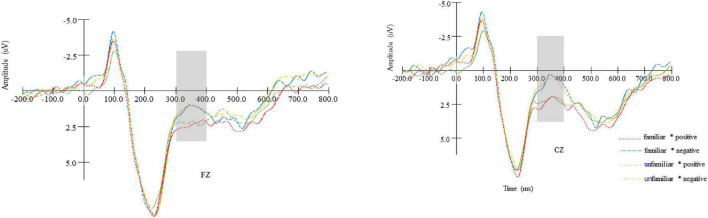
The N400 amplitude comparison of different combinations for brand familiarity (familiar vs. unfamiliar) and online reviews (positive vs. negative) in representative electrodes (FZ and FCZ).

The results of a 2 (brand familiarity: familiar vs. unfamiliar) × 2 (online review: positive vs. negative) × 9 (electrodes: F1, FZ, F2, FC1, FCZ, FC2, C1, CZ, and C2) repeated measures ANOVA indicated that brand familiarity and electrodes had no significant main effects (*ps* > 0.05), and therefore H3 was rejected. However, a significant main effect of online reviews was identified [*F*(1, 22) = 11.886, *p* < 0.05, η^2^ = 0.351], suggesting that a larger amplitude of N400 was elicited in the case of negative reviews (*M*_negative_ = 1.191, *SE* = 0.624) than that of positive reviews (*M*_positive_ = 2.312, *SE* = 0.627), as seen in Figure 4. Therefore, H4 was supported. However, the interaction effect of brand familiarity and online reviews was not significant (*p* > 0.05), and therefore H5 was rejected. Several interaction effects were again not significant: between brand familiarity and electrode point (*p* > 0.05); between online reviews and electrode point (*p* > 0.05); and between brand familiarity, online reviews, and electrode point (*p* > 0.05). The results of hypothesis testing seen in Table 3.

**TABLE 3:** Hypothesis testing results.

Hypothesis	Results
H1: The P200 amplitude of familiar brands is smaller than that of unfamiliar brands.	Supported
H2: The LPP amplitude of familiar brands is larger than that of unfamiliar brands.	Supported
H3: The N400 amplitude of unfamiliar brands is larger than that of familiarity brands.	Not supported
H4: The N400 amplitude of negative online reviews is larger than that of positive online reviews.	Supported
H5: There is an interaction effect between the online review and brand familiarity on N400 amplitude.	Not supported

## Conclusion and discussion

### Conclusion

With the help of the ERP approach, this research investigates the neural mechanism of consumers’ online booking decisions that are influenced by brand familiarity (familiar vs. unfamiliar) and online reviews (positive vs. negative). The main conclusions are as follows:

The behavioral results indicated that subjects had a higher booking rate in the positive reviews condition than that of negative reviews. However, we didn’t find a significant main effect of brand familiarity on booking rate, while the failure at behavioral level does not mean that it has no impact on the cognitive process before final decision-making (Wang et al., 2022). In fact, behavioral data are less sensitive than ERP data (Kenemans and Kähkönen, 2011). At the brain level, we found the P200 amplitude varied significantly between familiar and unfamiliar brands, and LPP amplitude also showed significant differences, which demonstrated that the cognitive activities related to the processing of familiar and unfamiliar brands were different. This is an interesting finding and also indirectly explains why the decision-making process of consumer’s needs to be explored by neuroscience methods instead of self-report. In addition, it was found that both brand familiarity and online reviews had significant main effects on response time, suggesting that consumers prefer to spend more time processing familiar brands than unfamiliar brands.

The ERP results showed that the familiar brands elicited a smaller P200 amplitude and a larger LPP amplitude than unfamiliar brands (e.g., Figures 2, 3). P200 is an early ERP positive-going component that reflects automatic and unconscious attention (Olofsson et al., 2008), with a larger P200 amplitude indicating more attentional resources in the early stage (Ma et al., 2018; Alzueta et al., 2019). LPP is a late ERP positive-going component, which reflects the later and conscious attention and time-specific emotional response (Herring et al., 2011). A larger amplitude of LPP is associated with more conscious attention and more emotional response (Eimer et al., 2012). It can be concluded that before final booking decision-making, the cognitive processing of brands can be divided into two stages: the early unconscious stage and the later conscious stage. In the early stage, consumers will pay more unconscious and automatic attention to unfamiliar brands (Ozkara and Bagozzi, 2021). With further development of processing, the past experience and knowledge (i.e., direct consumption experience and indirect information contact) in the consumers’ brains will be activated, and thereby they will focus their attention on the information of familiar brands.

Regarding the N400, its amplitude of negative online reviews was larger than that of positive online reviews (e.g., Figure 4). This finding was consistent with the result of linguistic research that negative words elicited a larger N400 amplitude than positive and neutral words (Herbert et al., 2008; De Pascalis et al., 2009). The reason is that negative words reflects a greater semantic activation (Kanske and Kotz, 2007) that could promote consumers to make a booking decisions. This also explains why the response time to negative online reviews is shorter than that to positive online reviews. Furthermore, the finding is also consistent with the research conducted by traditional self-report approaches which believed that negative reviews are more useful than (Casaló et al., 2015b; Rouliez et al., 2019; Roh and Yang, 2021) than positive reviews in the aspect of promoting consumers to exclude some products and choose other products.

However, we did not discover the main effect of brand familiarity on N400 and the interaction effect of brand familiarity and online reviews. Two reasons may explain why brand familiarity did not influence N400. For one thing, although some studies argue that N400 reflects the acceptable of exposed information (Jin et al., 2015; Zhang et al., 2019a), it may most focuses on violations and conflicts in essence (Huang et al., 2014). From this point of view, although a familiar brand may be more acceptable than an unfamiliar brand, it doesn’t mean that an unfamiliar brand is more violated and conflicted than a familiar brand. For other, research on familiarity in ERP research is typically associated with the “old-new effect” studied by the “learning-recognition” paradigm and reflected by FN400 and sometimes even N400 (Stróżak et al., 2021; Sánchez-Mora and Tamayo, 2021). Specifically, subjects are expected to study new words or pictures in the learning stage, and recognize them in the recognition stage. However, since the aim of the present work is different from that of the research on memory (the old-new effect), we did not design the learning-recognition process, so the difference between familiar and unfamiliar brands may not be successfully activated.

There are two possible reasons why the interaction between brand familiarity and online reviews was not observed. First, online reviews belong to high-scope cue with higher diagnosticity than brand familiarity (Wen et al., 2021). Online reviews alone will help consumers make booking decisions, and therefore consumers would not be affected by brand familiarity. Secondly, N400 component is sensitive to violations and conflicts (Huang et al., 2014). In the present study, the familiar-negative and the unfamiliar-negative stimuli elicited the largest and the second largest N400 amplitude in the four scenarios, respectively. The familiar-positive stimuli and the unfamiliar-positive evoked the penultimate smallest and the smallest N400 amplitude, respectively. Since a larger N400 amplitude indicates more conflicts, it is not difficult to posit that the fluctuation of N400 is caused by negative online reviews to a larger extent. That is, compared to brand familiarity, consumers’ booking decisions are mostly depended on online reviews.

### Theoretical implications

Booking decisions are an important topic in hospitality, while little (if any) researchers have centered on the underlying neural processing. To address this research gap, with the help of ERP, we discussed the cognitive process of consumers’ booking decisions based on brand familiarity and online reviews. The findings make several theoretical and practical contributions.

This study reveals an interesting and novel discovery: brand familiarity has no influence on the booking decisions of consumers, whereas it has a critical impact on their cognitive processing before the final decision-making. This finding is contrary to previous studies constructed by self-report measurement (McClure and Seock, 2020; Ruiz-Equihua et al., 2020; Wen et al., 2021). The possible reason is that self-report methods could only answer the “what” question—“what is the relationship between brand familiarity and booking decisions,” but couldn’t reveal the related neural processing, that is, the “how” question. In a nutshell, if researchers find that brand familiarity has no effect on booking decisions, it doesn’t mean that it could not influence booking decisions at all. Researchers should attempt to find a breakthrough in the neural processing.

Moreover, this work uncovers the complete cognitive process related to the effect of brand familiarity on booking decisions. Unfamiliar brands arouse more unconscious attention in the early stage, while familiar brands activate more conscious attention in the later stage. To our knowledge, little (if any) research, especially ERP research, explores the cognitive process related to brand familiarity. The present study fills the research gap in brand familiarity and ERP literature. In addition, this study showed that negative reviews elicited larger semantic conflicts reflected by a larger N400 amplitude, which provided neural evidence for previous research that demonstrated negative reviews were more useful than positive reviews. This finding deepens the understanding of negative reviews and enriches the literature on online reviews.

Furthermore, this work is one of only a few studies on the neural mechanism of consumers’ hotel booking decisions. Scant attention of previous research has been devoted to analyzing the neural activities of consumers’ booking decisions, which leads the public only know that brand familiarity, online reviews, or other product-related cues may impact consumers’ decisions (Chen and Chang, 2016; Luan et al., 2019; Wen et al., 2021) without knowing how and why. Based on P200, LPP, and N400, we attempt to open the “black box” in the brain of consumers to bridge the research gap and provide new research ideas.

Finally, this research also makes a major methodological contribution. Most of the existing research on online hotel booking used self-reported approaches, which are suitable for answering the “What” question (i.e., what is the relationship between product-related cues and online booking decisions) rather than the “How” question (i.e., how they impact booking decisions) (Wang et al., 2022). In addition, since decision-making is a sophisticated cognitive and psychological process, usually accompanied by implicit and unconscious neural activities (Zhang et al., 2019b), individuals may be unaware of their cognitive and psychological activities and unable to explain why they make such decisions. Therefore, neuroscience tools are recommended, such as ERPs, functional magnetic resonance imaging (fMRI), and positron emission tomography (PET), to open the “black box” in the brain of consumers related to decision-making.

### Practical implications

Some practical implications should also be considered. From the perspective of online reviews, the response time and the N400 amplitude under the condition of negative reviews were shorter and larger than that of positive reviews, respectively. That is, negative reviews will make consumers eliminate a product promptly. Therefore, preventing and coping with negative reviews is the top priority of the hotels rather than encouraging consumers to post positive reviews. To be specific, for the potential negative reviews when customers encounter service failure, hotels should immediately provide material and spiritual compensation to prevent consumers to from posting negative reviews. For the published negative reviews, hotels should apologize to the consumers through the internet or telephone and give compensations so as to get additional positive reviews from consumers.

In addition, a hotel with negative reviews should not only improve the quality of products and services as much as possible but also establish a normalization mechanism to prevent consumers from emotional conflicts caused by negative reviews. For instance, if consumers quickly leave the product page while browsing online reviews, which probably indicates that the consumers are affected by negative reviews, the online service personnel should immediately initiate a dialogue and timely alleviate the negative emotions caused by negative reviews through professional and high-quality introduction and communication.

From the perspective of brand familiarity, although it has no influence on the booking decisions of consumers, its effect on cognitive processing can not be ignored, which is the prerequisite of final decisions. In the present study, familiar brands elicited a smaller P200 and a larger LPP. Therefore, entrepreneurs should continue to make efforts to establish and improve brand familiarity so as to increase the popularity and influence of their hotels and attract consumers’ attention. For instance, hotels can invest more in social media marketing, including topics marketing and video marketing. Social media, such as Twitter, Instagram, Sina Weibo, and Tiktok, have a natural advantage in marketing, because of its timeliness, wide dissemination and huge user base. As long as there is mobile technology, there will be social media marketing. Once a post or video is on the hotlist of social media, it may be seen by tens of millions or even trillions of people, and the brand familiarity of the hotel can be improved promptly. In addition, hotels can also invite influencers to publicize their hotels. Influencers have a large number of loyal fans who prefer following their behavior, and enjoy interacting with them through “like,” “comment,” and “share,” and these interactions will be further interacted by the fans of these followers, and thus constantly improve the brand familiarity of hotels.

### Limitations

Despite the significance, this research also has some limitations. First, since the materials of ERP experiments have to be simple and short, the online hotel booking scenario during the experiment is generally abstract and easy than in the real life. Second, all the recruited subjects were college students. Due to COVID-19, it is difficult for us to reach general consumers. Subjects with various backgrounds should be involved in future research for a more comprehensive view of general consumers’ brain activities during online booking decisions. Third, we only consider the effects of brand familiarity and review valence. However, many other cues, such as price, sales, and product pictures, are also worthy of discussion. Finally, although our manipulation of brand familiarity was successful, some brands were fictitious. In future research, fictitious brands should be avoided.

## Data availability statement

The original contributions presented in this study are included in the article/supplementary material, further inquiries can be directed to the corresponding author.

## Ethics statement

The studies involving human participants were reviewed and approved by the Ethics Committee of the Neurotourism Laboratory at Huaqiao University. The patients/participants provided their written informed consent to participate in this study.

## Author contributions

PF and JW made substantial contributions to the work, participated in all aspects of the manuscript, conducted the experiment, analyzed the data, and wrote the manuscript. XD participated in the data acquisition and data interpretation stage. CL and FG oversaw the study and managed every part of the research. XYD revised the section of data analysis. All authors contributed to the article and approved the submitted version.
